# Telehealth Diabetes Prevention Program for Adults With Prediabetes in an Academic Medical Center Setting: Protocol for a Hybrid Type III Trial

**DOI:** 10.2196/50183

**Published:** 2023-11-13

**Authors:** Abigail Gamble, Tamkeen Khan, Alejandro Hughes, Yan Guo, Siga Vasaitis, Josie Bidwell, Brian Christman

**Affiliations:** 1 Department of Preventive Medicine John D Bower School of Population Health University of Mississippi Medical Center Jackson, MS United States; 2 Myrlie Evers-Williams Institute for the Elimination of Health Disparities John D Bower School of Population Health University of Mississippi Medical Center Jackson, MS United States; 3 American Medical Association Chicago, IL United States; 4 Optum Eden Prairie, MN United States; 5 Center For Informatics and Analytics University of Mississippi Medical Center Jackson, MS United States; 6 Department of Preventive Medicine School of Medicine University of Mississippi Medical Center Jackson, MS United States; 7 Department of Data Science John D Bower School of Population Health University of Mississippi Medical Center Jackson, MS United States

**Keywords:** prediabetic state, prevention, preventive medicine, Centers for Disease Control and Prevention, Medicare, telemedicine, telehealth, behavioral science, implementation science, implementation, research evaluation, cost-benefit analysis, diabetes, diabetic, cost, costs, economic, prevention, design, engagement, use, distant learning

## Abstract

**Background:**

Diabetes is a costly epidemic in the United States associated with both health and economic consequences. These consequences can be mitigated by participation in structured lifestyle change programs such as the National Diabetes Prevention Program (DPP) led by the Centers for Disease Control and Prevention. Mississippi consistently has among the highest rates of diabetes and prediabetes nationally. Implementing the National DPP through large health care systems can increase reach and accessibility for populations at the highest risk for diabetes. Translational research on the National DPP in Mississippi has not been studied.

**Objective:**

This study aims to evaluate the implementation and impact of the National DPP delivered using telehealth modalities at the University of Mississippi Medical Center in Jackson, Mississippi.

**Methods:**

An effectiveness-implementation hybrid type III research design is proposed. The study design is guided by the Reach, Effectiveness, Adoption, Implementation, and Maintenance framework and the Practical, Robust Implementation and Sustainability Model. Participants are being recruited via provider referral, and the DPP is being delivered by trained lifestyle coaches. Study participants include adult (≥18 years) patients eligible for the DPP with at least 1 encounter at 1 of 3 ambulatory clinic specialties (lifestyle medicine, family medicine, and internal medicine) between January 2019 and December 2023. The National DPP eligibility criteria include a BMI ≥25 kg/m^2^ and hemoglobin A_1c_ between 5.7% and 6.4%. The University of Mississippi Medical Center criteria include Medicare or Medicaid beneficiaries. The University of Mississippi Medical Center’s a priori implementation plan was developed using the Consolidated Framework for Implementation Research and includes 23 discrete strategies. The primary aim will use an embedded mixed method process analysis to identify and mitigate challenges to implementation. The secondary aim will use a nonrandomized quasi-experimental design to assess the comparative effectiveness of the DPP on health care expenditures. A propensity score matching method will be implemented to compare case subjects to control subjects. The primary outcomes include patient referrals, participant enrollment, retention, engagement, the incidence of diabetes, and health care resource use and costs.

**Results:**

At baseline, of the 26,151 patients across 3 ambulatory clinic specialties, 1010 (3.9%) had prediabetes and were eligible for the National DPP. Of the 1010 patients, more than half (n=562, 55.6%) were aged 65 years or older, 79.5% (n=803) were Medicare beneficiaries, 65.9% (n=666) were female, and 70.8% (n=715) were obese.

**Conclusions:**

This is the first translational study of the National DPP in Mississippi. The findings will inform implementation strategies impacting the uptake and sustainability of the National DPP delivered in an academic medical setting using distance learning and telehealth modalities.

**Trial Registration:**

ClinicalTrials.gov NCT04822480; https://clinicaltrials.gov/study/NCT03622580

**International Registered Report Identifier (IRRID):**

DERR1-10.2196/50183

## Introduction

### Background

Type 2 diabetes mellitus (herein referred to as diabetes) is a costly epidemic in the United States responsible for significant morbidity and mortality. In 2019, over 37.1 million (14.7%) adults (≥18 years) were diagnosed with diabetes [1]. Diabetes is a metabolic derangement characterized by insulin insensitivity resulting from insulin resistance, reduced insulin production, and eventual pancreatic beta-cell failure, leading to hyperglycemia. Prediabetes is often a precursor to diabetes, a condition with plasma blood glucose levels higher than normal (hemoglobin A_1c_ [HbA_1c_] 5.7%-6.4%) but not high enough to be characterized as diabetes (HbA_1c_ ≥6.5%) [2]. An estimated 96 million (38%) adults had prediabetes in 2019 [1].

In 2017, diabetes was estimated to impose a US $327 billion annual economic burden, including US $237 billion in direct medical costs [3]. A retrospective study of claims data from 2010 to 2014 among a commercially insured adult (18 to 64 years) population showed patients with newly diagnosed diabetes spent US $10,000 or more than their nondiabetic counterparts over the 5 years leading up to the diagnosis [4]. Annual medical expenditures are nearly one-third higher for those who subsequently develop diabetes relative to those who do not transition from prediabetes to diabetes, with an average difference of US $2671 per year [5]. At that cost differential, the 3-year return on investment (ROI) for diabetes prevention was estimated to be as high as 42% [5].

In 2010, the Centers for Disease Control and Prevention (CDC) introduced the National Diabetes Prevention Program (DPP) to mitigate the transition from prediabetes to diabetes [6-8]. In 2018, the Center for Medicare and Medicaid Services launched the Medicare DPP offering an unprecedented opportunity to reach the estimated 48.3% (25.9 million) of older adults with prediabetes [9]. Despite macrolevel policy support for disseminating lifestyle change programs (LCPs) such as the Medicare DPP, significant challenges hinder translation. There are gaps in program accessibility and population reach and challenges to community and organizational uptake and sustainability of LCPs [9-15]. A recent review by Ackermann and O’Brien [16] found that more than 1500 organizations had reached about 300,000 people with the DPP nationally, representing a mere 1% of the target population. In another review of Medicare DPP supplier organizations, Ritchie and Gritz [17] found 126 unique suppliers across 601 sites nationally (1 site per 100,000 beneficiaries), and the estimated reimbursement to cover program delivery was US $661 per beneficiary. The National DPP is less available to residents in rural locales [18], in counties with higher diabetes incidence, and among socioeconomically disadvantaged groups relative to their respective counterparts [19]. Furthermore, program attrition rates are the highest among racial and ethnic minority populations [14]. Efforts are needed to increase the overall detection, diagnosis, and referral of patients with prediabetes to an LCP such as the DPP, and tailored approaches for vulnerable populations are needed to ensure equity.

Hybrid implementation-effectiveness trials are particularly well suited to identify strategies to address these gaps. Blended designs study the translation of evidence-based interventions within a real-world context to foster more rapid translation over conducting these studies independently [20]. Curran et al [21] proposed 3 types of hybrid designs. Type I designs prioritize effectiveness testing and, secondarily, the gathering of contextual information for implementation. In type II designs, clinical and implementation aims are tested simultaneously, and type III designs prioritize implementation. Type III designs are specifically recommended when implementing an evidence-based practice or program such as the DPP in a new setting or population. Damschroder et al [10] used a type III design to study the implementation of LCPs to veterans through the Veterans Health Administration while comparing the effectiveness of the National DPP with the MOVE! weight management program [10]. The findings demonstrated that large health care systems have the potential to fill gaps in advancing equity in diabetes prevention through improved reach among priority populations and support for uptake in large health care systems. Critical implications included the need for buy-in among systems and local leaders, disseminating evidence-based findings to referring clinicians who may question the overall effectiveness of LCPs for diabetes prevention, and allocating sufficient time and resources for staff training. Conducting hybrid trials in regions with the highest rates of diabetes and disparities is a priority; however, in states such as Mississippi, where diabetes rates are persistently among the highest, no translational studies of the National DPP have been conducted.

### Aims

The primary aim of this study is to evaluate the implementation of the National DPP and Medicare DPP LCPs in 1 large health care system. The RE-AIM (Reach, Effectiveness, Adoption, Implementation, and Maintenance) framework [22] will guide a mixed methods process evaluation to understand how and why the DPP LCP worked, for whom, in what settings, and at what intensities. The secondary aim is to use a nonrandomized quasi-experimental design to assess the comparative effectiveness of the DPP on reducing diabetes risk, health care cost savings, and health care use. An overview of the study research questions is provided in Table 1.

**Table 1:** Research questions by measures of the Reach, Effectiveness, Adoption, Implementation, and Maintenance framework.

Measure		Research question
Reach		What percent of potentially eligible patients were excluded and participated? How representative were the participants of the target population?
Effectiveness		What impact did the intervention have on all participants who began the program?
Adoption		What percent of clinical sites and providers participated in the intervention?
Implementation		To what extent were the intervention components delivered as intended?
**Maintenance**		
	Organizational	To what extent was the intervention continued? How were the implementation strategies adapted? To what extent was the intervention modified? Can the institution maintain delivery of the LCP^a^? What is the cost-benefit of delivering versus not delivering the LCP to patients with prediabetes?
	Participant	What was the attrition rate? What was the representation of participants not completing the intervention? How did attrition impact conclusions about effectiveness?

^a^LCP: Lifestyle Change Program.

## Methods

### Study Design

This study uses a hybrid type III research design [21] and follows the Standards for Reporting Implementation Studies (StaRI) [23]. StaRI guidelines were developed to promote the translation of implementation studies. The 27-item checklist ensures the transparent reporting of implementation strategies and the effectiveness of the intervention that is being implemented. The proposed study will be the first to comprehensively evaluate the translation of the National DPP and Medicare DPP in Mississippi.

### Ethics Approval

This study was reviewed by the institutional review board at the University of Mississippi Medical Center (2020V0327). This trial is registered with ClinicalTrials.gov (NCT04822480).

### Implementation Context

#### Setting

Mississippi is consistently ranked among the states with the highest rates of diabetes (n=326,420, 14.4%) and prediabetes (n=814,000, 35.6%) in the United States [24]. It is also the only state where every county is included in the Diabetes Belt, a cluster of counties in the Southeastern United States where obesity and physical inactivity account for one-third of all diagnosed diabetes cases [25]. Mississippi has the highest percentage of Black (n=1,111,340, 37.8%) populations of any state in the United States and persistently has higher rates of poverty (n=570,371, 19.4%) and a lower median household income (US $49,111) compared to national statistics (n=38,661,356, 11.6% and US $69,021, respectively) [26]. The exceedingly high rates of poverty contribute to Mississippi having the highest Federal Medical Assistance Percentage in the nation (77.27%) [27]. It is the nation’s fourth most rural state [20], where 65 (79.3%) of the 82 counties are considered rural areas. According to the Health Resources and Services Administration [28], every one of Mississippi’s 82 counties is designated a medically underserved area [28].

#### Implementation Site

The University of Mississippi Medical Center (UMMC) in Jackson, Mississippi, is the state’s only academic medical center. Annually, UMMC physicians conduct over 535,000 clinical visits; 195,000 outpatient and 135,000 emergency room encounters; and 31,000 hospital admissions serving pediatric and adult patients statewide. The UMMC is a minority-serving and research-intensive health care organization with a state mandate to provide no less than 50% of its services to indigent persons (Miss. Code Ann. § 37-115-27; 2017).

The UMMC is also home to 1 of only 2 telehealth centers of excellence in the country funded by the Health Resources and Services Administration (6-U66RH31459-04-01). In partnership with telehealth, the UMMC launched its DPP program in 2020. Implementing the National DPP LCP through health care systems, such as the UMMC, has the potential to increase program reach and accessibility for residents who are among those at the highest risk for diabetes in the most medically underserved communities; however, no studies of the translation of the DPP have been conducted in Mississippi. The proposed study will be the first to comprehensively evaluate the translation of the National DPP and Medicare DPP in Mississippi.

#### Implementation Strategy

The implementation plan was developed using the CFIR (Consolidated Framework for Implementation Research) [29]. The CFIR is a determinants framework for navigating the contextual factors that affect the uptake of innovations by health care institutions. The framework is comprised of 39 constructs across 5 complex interacting domains such as intervention characteristics, organizational inner setting context, outer setting context, characteristics of individuals involved with implementation, and implementation processes. The 4 interrelated processes include planning, engaging, executing and reflecting, and evaluating. The processes interact dynamically to achieve organizational (eg, UMMC health care system) and individual (eg, UMMC clinical providers) level use of an intervention as intended (eg, use of electronic health record system for screening and referral of patients at risk for diabetes). The UMMC’s a priori implementation plan includes 23 [24] discrete strategies described in Table 2 [30]. One strategy is taking a phased approach to implementation. Thus, in phase 1 of implementation, 3 clinical specialties were targeted based on provider buy-in including family medicine (n=8 clinical sites), lifestyle medicine (n=1 clinical site), and internal medicine (n=5 clinical sites).

**Table 2:** Discrete implementation strategies according to the Consolidated Framework for Implementation Research domains and taxonomy.

Domain		Strategy
**Implementation processes**		
	Evaluative and iterative strategies	Conduct a needs assessment to support the need for diabetes prevention LCP^a^ Develop a formal blueprint that defines goals and strategies Access various organizational aspects to determine their degree of readiness to implement, barriers that may impede implementation, and strengths that can be used in the implementation effort Stage implementation scale-up by starting with demonstration projects and gradually moving to a system-wide rollout Collect and summarize clinical performance data to monitor, evaluate, and modify provider behavior Obtain and use patient feedback on LCP to enhance program use and uptake
	Adapt and tailor to context	Involve, hire, and consult experts to inform management on the use of data generated by implementation efforts Tailor strategies to address barriers and leverage facilitators
**Inner setting**		
	Financial strategies	Secure external funding to support implementation and dissemination Explore innovative models of bundled payments
	Change infrastructure	Change and adapt the location of clinical service sites to increase access Change records systems to allow better assessment of implementation or clinical outcomes Develop and implement tools for quality monitoring
**Individuals**		
	Develop relationships	Identify early adopters to learn from their experiences Inform providers identified as opinion leaders to serve as DPP^b^ Champions, influence other providers, and contribute to an organizational climate that values prevention through lifestyle change
	Provider engagement	Meet with providers in their practice settings to teach them about the DPP with the intent of increasing referrals to the DPP Plan for and conduct continuous training Develop and disseminate informational materials to educate about the DPP and how they can refer their patients
	Support clinicians	Create a new clinical team by adding an operations manager and integrating trained lifestyle coaches into the clinical team Develop reminder systems designed to help clinicians recall information and prompt them to use the clinical innovation
**Outer setting**		
	Patient engagement	Develop strategies with patients to encourage adherence and problem-solving barriers Prepare patients to be active participants in preventing diabetes
	Develop multilevel and interdisciplinary partnerships	Identify and strengthen external partnerships and collaborations centered on diabetes prevention

^a^LCP: Lifestyle Change Program.

^b^DPP: Diabetes Prevention Program.

### Intervention Characteristics

#### National DPP

The National DPP is a 12-month LCP delivered in a group setting by a trained lifestyle coach. The core 16 sessions are delivered weekly in the first 6 months of the program. In the maintenance phase, a minimum of 6 sessions are delivered monthly. The evidence-based program aims to develop behavior change skills leading to increased physical activity (150 minutes per week) and modest weight loss (7% reduction in total body weight), which are proven to prevent or delay diabetes in adults [7]. The DPP LCP is a clinical service of lifestyle medicine in the Department of Preventive Medicine in both the School of Medicine and John D. Bower School of Population Health at the UMMC. The LCP achieved full recognition from the CDC within the first year and, in 2020, became the third Medicare-approved DPP provider in Mississippi.

#### Interventionists

Lifestyle coaches are UMMC employees and include registered dietitians, project coordinators in population health, and community health workers. All interventionists completed a CDC-recognized lifestyle coach training, have a registered National Provider Identifier, and completed continuing education units. Newly trained lifestyle coaches shadow an experienced coach prior to leading an independent cohort of patients.

#### Delivery

Group-based sessions are delivered using BigBlueButton, an open-source web-based classroom software [31]. This educational platform provides a unique link to connect participants using videoconferencing. Participants click a homeroom link, type their names, and enter the room. Weekly weight is tracked using digital body weight scales (developed by BodyTrace, Inc) equipped with cellular connectivity. Weekly physical activity minutes are reported to the lifestyle coach via phone call or email before or immediately following each session. Participant attendance, weight, physical activity, and billing (Medicare-eligible participants only) are tracked in patients’ electronic medical records in Epic (Epic Systems Corporation).

#### Eligibility Criteria

UMMC adult patients (≥18 years) who are overweight (BMI≥25 to 29.9 kg/m^2^) or obese (BMI≥30 kg/m^2^), have an HbA_1c_ between 5.7% and 6.4%, and are Medicare or Medicaid beneficiaries are recruited for the LCP by physician electronic referral. Using the Healthy Planet coordinated care management tool in Epic [32], physicians are prompted at the patient encounter by Best Practice Alert to diagnose, inform, and refer patients with prediabetes to the LCP. Epic is a patient-centered, cloud-based electronic health record software program comprised of multiple systems to facilitate patient care. Patients with 1 or more of the following are excluded: <18 years; pregnant or planning to become pregnant within the next 12 months; a primary or secondary diagnosis of diabetes, including all International Classification of Diseases, Tenth Revision (ICD-10) codes with a prefix of E11; or end-stage renal disease (ICD-10 code N18.6). Patients with a diabetes diagnosis and an HbA_1c_ value above 6.4% will be excluded.

Given that the LCP is delivered in a telehealth setting, it is preferred that patients have their own computer, tablet, or smartphone equipped with a camera and connected to a reliable Wi-Fi network. However, in a largely rural and underresourced state, this is not a realistic expectation. Thus, participants are eligible to participate if they own or have access to a computer, tablet, or smartphone connected to a reliable Wi-Fi network, have a mobile or landline phone to call into group sessions, or agree to use a study-provided tablet equipped with Wi-Fi and cellular connectivity. Study-provided tablets have restricted use and are provided to participants for the duration of the LCP and must be returned to the study team at the end of the program.

#### Recruitment, Retention, and Engagement

Electronic referrals to the LCP were extracted from Epic monthly between 2019 and 2021. In 2022, a dedicated LCP staff person was hired, and referrals have since been extracted from Epic twice weekly. Prior to making contact, the coordinator confirms the patient’s eligibility. Medical records of ineligible patients are updated, and they are referred to the Lifestyle Medicine clinic rather than being contacted. Among those eligible, up to 3 attempts are made to contact each patient including phone calls, voicemails, and emails. The date, time, type and outcome of contact, and the staff person’s name are documented. At a failed third attempt to make contact, the contact attempt details are recorded in the patient’s medical record and the referral is closed. Patients declining participation are also noted in their respective medical records and the referral is closed. Interested patients are added to a cohort and provided with information to attend session 0, an informational session.

Following session 0, interested participants are enrolled in the LCP and begin session 1 the following week. Scheduling cohorts consider patient preference and lifestyle coach availability. All session dates over the 12-month intervention period are determined prior to session 0, and participants receive the 12-month schedule at the start of the program. Makeup sessions are provided as needed but participants are encouraged to attend regularly scheduled group sessions. There is no limit to the number of participant makeup sessions. Up to 2 reminder contacts are made with each participant prior to each session. All participants with an email address are contacted via email, followed by a reminder phone call at which point participants are asked to report their weekly weight and physical activity minutes. For participants who do not have an email address, up to 2 phone call contacts are made. These procedures are consistent across participants and cohorts. In addition, when a participant misses 2 consecutive sessions, the project coordinator attempts to make contact to offer support in overcoming barriers and re-engaging them in the program. After 4 consecutive missed sessions and failed attempts to make contact, the participant is excluded from the program.

### Implementation Outcomes

#### Overview

Implementation outcomes are mapped across the 5 domains of the RE-AIM framework (Table 2). The evaluation seeks to determine the intervention reach among patients at risk for diabetes, clinic and provider adoption, implementation fidelity, intervention effectiveness in preventing diabetes, and the organizational and patient uptake of the intervention. Implementation outcomes, research questions, measures, and methods are outlined in Table 3. Data sources will include electronic medical records, session notes provided by lifestyle coaches, intervention cohort and delivery details, meeting notes, and evaluation of DPP sessions conducted by an external reviewer.

**Table 3:** Implementation outcomes following the Reach, Effectiveness, Adoption, Implementation, and Maintenance framework.

Outcomes and measure			Methods
**Reach**			
	Eligibility	Number and proportion of patients eligible among the total number of patients in an ambulatory setting	
	Enrollment	Number and proportion of patients enrolled among the total number of referred patients Descriptive characteristics of patients enrolled	
	Exclusion	Number and proportion of patients excluded among the total number of patients referred Descriptive characteristics of patients excluded Reasons why patients are excluded	
	Conversion rate	Number and proportion of patients referred, eligible, and enrolled among those referred and eligible	
**Effectiveness**			
	Incidence of diabetes	The ratio of the number of diabetes cases to the total time at risk among the LCP^a^ participants compared with control-matched nonparticipants	
**Adoption**			
	Clinical sites	Number and proportion of clinical sites making referrals among sites eligible to make referrals	
	Providers	Number and proportion of providers making referrals among providers eligible to make referrals The mean number of referrals made among the top 3 providers making referrals	
**Implementation**			
	Fidelity	Organizational recognition status awarded by the CDC^b^ Barriers, solutions, and facilitators to program delivery Observation of randomly selected DPP^c^ sessions	
	Intervention costs	Microcosting: time, training, and program materials	
**Maintenance**			
	Organizational	Number of new clinical sites making referrals Number of new providers making referrals	
	Participant engagement	Number and proportion of sessions attended	
	Participant retention	Duration of participation in days from first to last session within the 12-month program Number of patients completing the 12-month LCP compared to the number of patients enrolled	

^a^LCP: Lifestyle Change Program.

^b^CDC: Centers for Disease Control and Prevention.

^c^DPP: Diabetes Prevention Program.

#### Reach

The extent to which the target population is exposed to the intervention or population reach will be determined by the number of eligible patients referred to and enrolled in the LCP, yielding the appropriate referral to enrollment conversion rate. The a priori conversion rate goal is 50%. Reasons for exclusion and participant refusal to participate will be documented where possible. Descriptive characteristics such as age, sex, race, ethnicity, and BMI be explored and compared between patients eligible for the program who were referred and not referred.

#### Effectiveness

The LCP effectiveness will be determined by the differential incidence of diabetes. Diabetes will be defined by diagnosis code or HbA_1c_ values greater than 6.4%. Data will be extracted from patient medical records and reviewed biannually. The a priori reduction in diabetes diagnosis among the intervention compared with the control group is 50%.

#### Adoption

The adoption of the LCP will be defined as the number and proportion of clinical sites with referring providers making referrals to the LCP, and the number and proportion of eligible providers making referrals. Data will be extracted from medical records and reviewed quarterly. The a priori adoption clinical and provider addition rates are 70%.

#### Implementation

Implementation fidelity and costs will be evaluated. Fidelity will be based on the UMMC’s recognition category by the CDC. The CDC standards consider participant recruitment, retention, attendance, the proportion of participants meeting the LCP 7% weight loss goal, the proportion of participants achieving 150 minutes or more of physical activity per week, and reductions in HbA_1c_. Barriers, solutions, and facilitators will be documented by the project coordinator and lifestyle coaches in a weekly session log, during quarterly lifestyle coach meetings, and in strategic planning meetings with UMMC leadership and the American Medical Association Improving Health Outcomes team.

#### Maintenance

Ongoing organizational and participant engagement in the LCP will provide a robust measure of program maintenance. Longitudinal changes in the number of clinical sites and providers making referrals will be extracted from Epic quarterly, including data to monitor referral maintenance across sites and providers. Attendance and retention in the LCP will provide a measure of maintenance among participants. The a priori participant retention rate is 60%.

### Economic Outcomes

The secondary aim of this study is to use a nonrandomized quasi-experimental design to assess the comparative effectiveness of the LCP on cost savings. The primary outcome will be health care expenditures (Table 4). For economic measures, trends in per capita medical expenditures will be tracked among a panel of case (LCP participant) and control (non-LCP participant) subjects. To compare variations in spending, the differences in the arithmetic mean, compound annual growth rates, and propensity score matching models will be implemented to compare case and control subjects. The investigators may also record data from those with prediabetes who are later diagnosed with diabetes and individuals with prediabetes who are not later diagnosed with the condition. These estimates of spending differentials and evidence of DPP participation and engagement rates will further enhance the estimates of potential cost savings and ROI if diabetes is prevented or delayed in the at-risk population who participate in the LCP.

**Table 4:** Economic outcomes.

Outcomes and measure			Method
**Health care use**			
	Medical encounters	Number and type of medical encounters extracted from electronic medical records Types of CPT^a^ codes at each medical encounter extracted from electronic medical records	
**Medical cost**			
	Medical expenditures	Dollar amount billed per encounter	
	Condition or problem	ICD-10^b^ code or CPT codes per encounter	

^a^CPT: Current Procedural Terminology.

^b^ICD-10: International Classification of Diseases, Tenth Revision.

### Allocation

#### Overview

Study participants are identified in Epic using the eligibility criteria. Patients are placed in the intervention or comparison group based on natural selection. Each participant is assigned a study identification number that is matched to their medical record number. There is no interaction between study personnel and participants.

#### Intervention Group

The intervention group includes eligible patients referred to and enrolled in the LCP. Once a participant is enrolled in the study, they are assigned a study identification number.

#### Comparison Group

The comparison group includes patients meeting the inclusion criteria who are not enrolled in the LCP. Once a patient is enrolled in the DPP, they are no longer included in the control group. These participants include patients with and without a referral. Patients referred to the LCP, deemed ineligible based on insurance type, and referred to lifestyle medicine are excluded from the comparison group. Participants in the comparison group are matched to their intervention counterparts for final effectiveness and cost-benefit analyses. Participants enrolled in the comparison group are assigned a study identification number.

### Study Period

The baseline period is January 2019 to December 2019. The study period is from September 2020 (the start of the first DPP patient cohort) through December 2023.

### Statistical Analyses

#### Implementation Analysis

An embedded mixed method process analysis is being conducted to identify and mitigate challenges to implementation. Quantitative data are extracted from Epic and reviewed quarterly to inform reach and adoption. Quarterly data are used to adapt questions to further understand the quantitative data using qualitative methods. Qualitative data are subsequently analyzed using a narrative realist analytic approach. Meeting minutes, observation notes, and lifestyle coach feedback are compiled and analyzed in Word (Microsoft Corp) documents twice annually. Solutions to challenges are framed in the context of what is acceptable and appropriate to patients and providers and what is feasible within the context of the health care system (inner setting) and outer setting conditions, policies, and funding [33]. Findings are used in the ongoing planning process to continuously adapt, implement, and evaluate. Similar complex mixed methods designs have been used for evaluating the implementation of lifestyle interventions within new populations and settings [34-38].

#### Economic Analysis

Arithmetic means will be calculated overall and for specific sectors of health care between the 2 groups. Compound annual growth rates in per capita expenditures will be calculated to track the geometric progression ratio and estimate the rate of growth in spending time. A propensity score matching method will be implemented to compare case subjects to control subjects. The model takes the form of *P*(*X*)=*Pr* (*D*=1*jX*), where *D*=1 indicates DPP participation and matches on the probability of participation instead of attempting to create a match for each participant with the same value of *X*. This model aims to account for unobservables and obtain an unbiased and accurate measure of costs. Matched control subjects who do not participate in a DPP must be similar to the case subjects who did participate such that the only difference is attributed to the disease. The matching model in this analysis will control for the following: patient age; sex; race; ethnicity; zip code; and prevalence of other conditions such as but not limited to hypertension, chronic obstructive pulmonary disease, congestive heart failure, cancer, and metformin use in all years of the data. Differences in spending will be compared between individuals with prediabetes who participate in a DPP and those individuals who do not compute net savings and ROI (if any) of program participation. A similar methodology may be applied to compare individuals who progress to diabetes relative to those who do not progress for more in-depth cost savings and ROI analysis. Given this is the first time the DPP will be implemented at the UMMC, the sample size will be determined based on the convenience sample or the number of participants enrolled based on the proposed recruitment and retention strategies.

### Data Security and Quality Assurance

Data will be exported from Epic and stored in password-protected computerized files. All data are deidentified at extraction. Data files are stored on the UMMC secure server and shared with the American Medical Association via a secure file transfer process and password protection.

## Results

**Figure 1:**
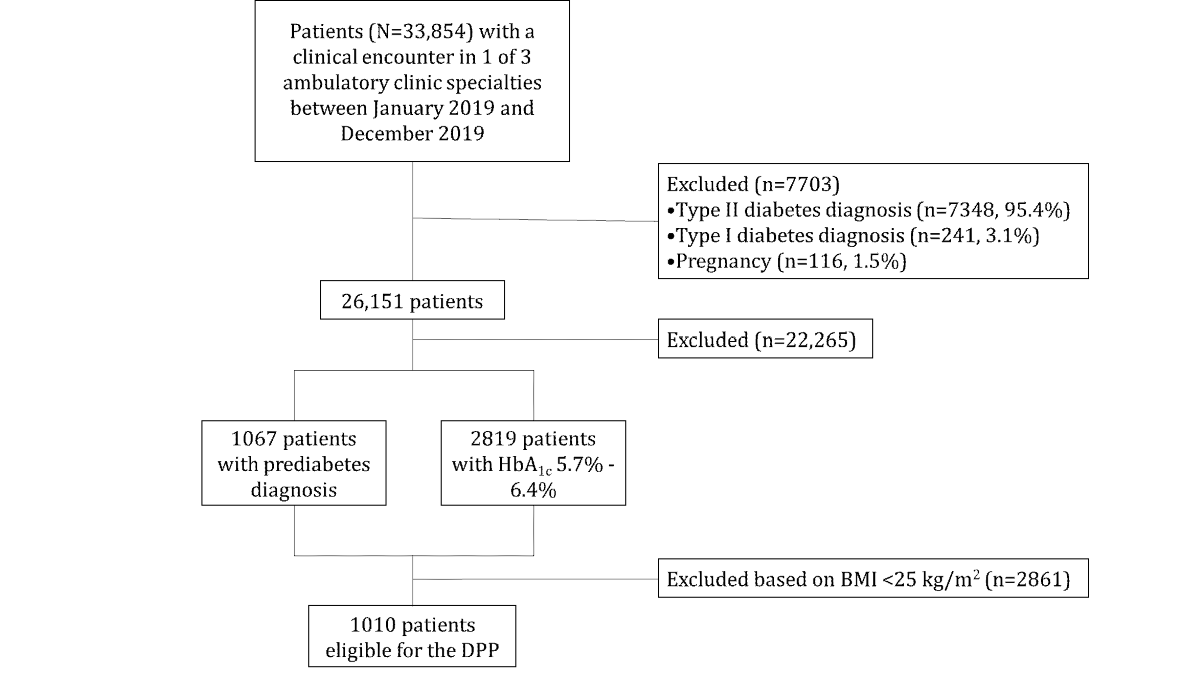
Baseline recruitment. DPP: Diabetes Prevention Program; HbA_1c_: hemoglobin A_1c_.

The baseline recruitment is depicted in a flow diagram in Figure 1. In 2019, a total of 33,854 adult patients had at least 1 clinic encounter at 1 of 3 UMMC specialty sites (lifestyle medicine, family medicine, or internal medicine). More than half (n=19,631, 58%) were non-Hispanic Black and 64.2% (n=21,744) were female. The mean HbA_1c_ was 6.4% (SD 1.69%), 49.7% (n=16,812) were obese, and 25.7% (n=8698) had a BMI between 25 and 30 kg/m^2^. About 1 in 5 patients had diabetes (n=7348, 21.7%) and 467 (1.4%) had end-stage renal disease.

Of the 33,854 patients, 7703 (22.7%) patients were excluded based on diabetes diagnosis (7348/7703, 95.4%), type I diabetes mellitus diagnosis (241/7703, 3.1%), or pregnancy (116/7703, 1.5%).

Among the remaining 26,151 patients, 1067 (4.1%) patients had a prediabetes diagnosis (n=1067, 4.1%), and 2819 (10.8%) had an HbA_1c_ between 5.7% and 6.4%. After applying the BMI eligibility criterion, 1010 (3.9%) patients were eligible for the National DPP. Baseline characteristics for those patients are described in Table 5. Future outcomes will be published in peer-reviewed implementation science journals and presented at national, regional, and state professional meetings and conferences.

**Table 5:** Baseline characteristics of patients eligible for the National Diabetes Prevention Program.

Characteristics		Values (n=1010)
**Age group (years), n (%)**		
	18-24	16 (1.6)
	25-44	104 (10.3)
	45-64	328 (32.5)
	≥65	562 (55.6)
**Sex, n (%)**		
	Female	666 (65.9)
	Male	344 (34.1)
**Race, n (%)**		
	Non-Hispanic Black	677 (67)
	Non-Hispanic White	302 (29.9)
	Other	31 (3.1)
Hemoglobin A_1c_, mean (SD)		5.93 (0.31)
**Weight status, n (%)**		
	Overweight	295 (29.2)
	Obese	715 (70.8)
**Insurance type, n (%)**		
	Medicare	803 (79.5)
	Medicaid	207 (20.5)

## Discussion

This is the first implementation study of the National DPP LCP in Mississippi. Iterative findings and adaptations will elucidate strategies to successfully improve access to the National DPP, improve awareness of prediabetes and LCPs, and meet physician and patient needs for diabetes prevention.

The hybrid study design is a strength of the proposed study. Limitations to the sample size as recruitment strategies are tested and refined may be a limitation of this study; however, outcomes from this study will be used to calculate a robust sample size for testing in phase 2 of implementation, which will include additional clinical sites and providers.

The implementation strategies from this study can be scaled and findings replicated to measure the impact of diabetes prevention efforts within other health care systems. In addition, given the relatively recent introduction of the Medicaid DPP and reimbursement models for Medicaid agencies, a longitudinal study, such as this, may be relevant to other large health systems seeking to quantify the impact of their diabetes prevention efforts in Medicare and Medicaid populations.

## Abbreviations

CDC: Centers for Disease Control and Prevention
CFIR: Consolidated Framework for Implementation Research
DPP: Diabetes Prevention Program
HbA1c: hemoglobin A1c
ICD-10: International Classification of Diseases, Tenth Revision
LCP: Lifestyle Change Program
PRISM: Practical, Robust Implementation and Sustainability Model
RE-AIM: Reach, Effectiveness, Adoption, Implementation, and Maintenance
ROI: return on investment
StaRI: Standards for Reporting Implementation Studies
UMMC: University of Mississippi Medical Center

## Data Availability

The data sets generated during this study are not publicly available due to their containing information that could compromise the privacy of research participants but are available from the corresponding author on reasonable request.
